# Supplementary material for: Evaluation of serum VIP and aCGRP during pulmonary exacerbation in cystic fibrosis: A longitudinal pilot study of patients undergoing antibiotic therapy
Source: PLoS One. 2023 May 5;18(5):e0284511. doi: 10.1371/journal.pone.0284511 (PMC10162560; doi:10.1371/journal.pone.0284511)
Supplement: Attachment — Submitted filename: Response letter.docx [file pone.0284511.s003.docx]

**Dear Editor,**

Further to our correspondence a couple of weeks ago, I’m attaching the revised version of my article entitled “Evaluation of serum VIP and aCGRP during pulmonary exacerbation in cystic fibrosis: a longitudinal pilot study of patients undergoing antibiotic therapy.” with Submission ID: PONE-D-22-25130R1.

We have edited the manuscript to address all the comments. I hope that the changes we’ve made resolve all the concerns about the article.

Thank you once again for your time and interest. I look forward to hearing from you.

**Sincerely,**

**Maha**

**Below please find our responses:**

**Reviewer #1:**

The authors of the PLOS ONE submission “Evaluation of serum VIP and aCGRP during pulmonary exacerbation in cystic fibrosis: a longitudinal pilot study of patients undergoing antibiotic therapy” have addressed my technical concerns regarding the use of multiple univariate comparisons. However, there remains an aspect of this submission (which I could not fully appreciate due to the previous analytical approach) that must be resolved.

**Response: A multivariate model with stepwise selection was used instead of the univariate model to test all variables and select those with significance without retesting or inflating alpha levels.**

**MAJOR COMMENTS:**
The authors engage in a form of circular reasoning regarding the utility of aCGRP and VIP measure as biomarkers of CF exacerbation response:
First, they state that “monitoring of cystic fibrosis is difficult especially in cases of pulmonary exacerbations where patients fail to regain the baseline pulmonary function despite standard treatment” (it’s not clear how a treating clinician can know that an individual has failed to regain baseline without monitoring… so clearly monitoring must be possible in this instance).
Then, they proceed to describe aCGRP and VIP changes during antimicrobial exacerbation treatment as a biomarker of treatment response without **methodologic description of how *clinical* responses to treatment were assessed**, recorded, or incorporated into analyses. They state that spirometry was either not available or uninformative and thus they relied on clinician evaluation… but I could not find these clinician assessments of response. How can the investigators conclude that aCGRP changes are indicative of treatment response without describing responses?

**Response: Thanks for your comment. We agree that the statement “Adequate monitoring of cystic fibrosis is difficult especially in cases of pulmonary exacerbations where patients fail to regain the baseline pulmonary function despite standard treatment” was not correct and we deleted it. We replaced the statement by the following: “Objective monitoring of improvement during treatment of pulmonary exacerbation can be difficulty in children when pulmonary function testing cannot be obtained.”**

**It was stated in the Method section lines 110-112 that the duration of treatment was 14 days, after which patients returned to baseline:**

**“The duration of antibiotic therapy for pulmonary exacerbation was 2 weeks for all patients, which is the duration of antibiotic therapy where patients returned to a symptomatic baseline”, and then we added the phrase “and had clinically improved based on clinician’s assessment” to make it clearer that the improvement was based on clinical response.**

**MINOR COMMENTS**
The authors can be more disciplined in their manuscript construction.

1. Lines 167 to 170 on page 9 of the results contain elements of Methods, Results and Discussion: “By analyzing the changes overtime of LOG VIP and aCGRP2 serum levels using repeated measures models, we investigated the factors responsible for these changes [a Method]. It was found that there were significant between-subjects effects over the LOG VIP [a Result], which indicates that the difference between the levels means which is not due to the time [a Discussion].”

**Response: The sentence “By analyzing the changes overtime of LOG VIP and aCGRP2 serum levels using repeated measures models, we investigated the factors responsible for these changes” has been shifted to the [methods] section, and the sentence “which indicates that the difference between the levels means which is not due to the time” has been shifted to the [discussion] section.**

2. Further, rather than state: “Figure 3 shows the LS means 171 and p-values for these effects” after this passage, why not just append the second sentence with “(Figure 3)”?

**Response: The paragraph has been rephrased.**

3. This manuscript is overly long at the expense of a clear description of observations. There is entirely too much discussion of the biology of aCGRP and VIP. A few sentences identifying and referencing key observations is more than adequate. Observations that these are interesting molecules are tangential to the matter at hand, which is how well they associate with exacerbation treatment.

**Response: Changes to the discussion has been performed where extra information about the molecular role of the markers has been deleted, and additional evidence of their role as potential biomarkers in CF has been discussed; lines 240-243 and lines 266-274.**

4. The figures are almost illegible. The fonts are too small, especially in Figures 3 and 4 and the point estimates for LS means in Figures 1 and 2 should have 95% CI or SE bars included.

**Response:** **The figures for all factors have been updated. for overall LS means, we have included the SE in tables instead of adding them to the figures.**
